# The Efficacy of a New AMCOP^®^ Elastodontic Protocol for Orthodontic Interceptive Treatment: A Case Series and Literature Overview

**DOI:** 10.3390/ijerph19020988

**Published:** 2022-01-16

**Authors:** Alessio Danilo Inchingolo, Assunta Patano, Giovanni Coloccia, Sabino Ceci, Angelo Michele Inchingolo, Grazia Marinelli, Giuseppina Malcangi, Valentina Montenegro, Claudia Laudadio, Chiara Di Pede, Mariagrazia Garibaldi, Zamira Kruti, Maria Elena Maggiore, Antonio Mancini, Ludovica Nucci, Ioana Roxana Bordea, Antonio Scarano, Felice Lorusso, Gianna Dipalma, Daniela Di Venere, Filippo Cardarelli, Francesco Inchingolo

**Affiliations:** 1 Department of Interdisciplinary Medicine, University of Bari “Aldo Moro”, 70124 Bari, Italy; ad.inchingolo@libero.it (A.D.I.); giovanni.coloccia@gmail.com (G.C.); angeloinchingolo@gmail.com (A.M.I.); graziamarinelli@live.it (G.M.); giuseppinamalcangi@libero.it (G.M.); valentinamontenegro@libero.it (V.M.); c.lauda@hotmail.it (C.L.); c.dipede1@studenti.uniba.it (C.D.P.); mgr.garibaldi@libero.it (M.G.); zamirakruti@hotmail.it (Z.K.); m.e_maggiore@yahoo.it (M.E.M.); dr.antonio.mancini@gmail.com (A.M.); giannadipalma@tiscali.it (G.D.); daniela.divenere@uniba.it (D.D.V.); drfilippocardarelli@libero.it (F.C.); 2 Multidisciplinary Department of Medical-Surgical and Dental Specialties, University of Campania Luigi Vanvitelli, Via L. De Crecchio 6, 80138 Naples, Italy; ludovica.nucci@unicampania.it; 3 Department of Oral Rehabilitation, Faculty of Dentistry, Iuliu Hațieganu University of Medicine and Pharmacy, 400012 Cluj-Napoca, Romania; 4 Department of Innovative Technologies in Medicine and Dentistry, University of Chieti-Pescara, 66100 Chieti, Italy; ascarano@unich.it

**Keywords:** interceptive orthodontics, eruption guidance appliance, elastodontics, elastodontic therapy, elastodontic appliances

## Abstract

Background: Elastodontics is a specific interceptive orthodontic treatment that uses removable elastomeric appliances. They are functional appliances that produce neuromuscular, orthopedic and dental effects. Thus, these devices are useful in the developmental age, when skeletal structures are characterized by important plasticity and adaptation capacity, allowing to remove factors responsible for malocclusions. Elastomeric devices are generally well tolerated by patients requiring simple collaboration and management. This work can be useful to update all orthodontists already adopting these appliances or for those who want to approach them for the first time. This study aimed to describe four cases treated with new elastomeric devices called AMCOP Bio-Activators and to provide an overview of elastodontics, its evolution, indications and limits. Methods: A total of four clinical cases were presented after a treatment period of 16–20 months to evaluate the clinical and radiological effects of the elastodontic therapy. Results: The effectiveness of Bio-Activators on clinical cases was evidenced with a significant improvement in skeletal and dentoalveolar relationship, and malocclusion correction in a limited treatment period (16–20 months). Conclusions: The Bio-Activators showed clinical effectiveness to achieve therapeutic targets according to a low impact on the patient’s compliance.

## 1. Introduction

In the twentieth century, the use of functional devices spread throughout Europe and then to many countries [1,2,3,4,5,6,7,8]. The main purpose of functional treatment is to “guide” the correct growth of the bone bases by stimulating the perioral muscles. When any muscular interferences or dentoskeletal malocclusions are treated at a very early age there is a greater guarantee that there is a stable balance between the jaws and the muscular component [9]. The ideal therapy is in the preschool child when the plasticity of skeletal structures makes the therapy fast and stable over time [10]. It has been suggested that interceptive orthodontic treatment allows the correction of developing problems in the mixed dentition in 15% of cases and their improvement in 49% [11]. The term elastodontics refers to a specific type of interceptive orthodontic treatment based on the use of removable elastomeric devices. These devices are characterized by an extreme simplicity in terms of use by the patient, by safety and by construction [12,13]. A new elastodontic approach is represented by AMCOP^®^ (Cranium-Occluded-Postural Multifunctional Harmonizers) Bio-activators (Micerium spa patent). These Bio-activators consist of two flanges, one vestibular and one lingual, which delimit a free central area. The absence of indentations allows simultaneous involvement of both dental arches with the repositioning multidimensional orthopedic effect and gives teeth freedom to find their position without any pressures. Furthermore, these devices act as rehabilitative therapy for altered muscular functions. The presence of a lingual ramp and a button for the tongue, in addition, allows the restoration of a correct posture and lingual function. Several devices are available for the different types of malocclusions, distinguished by different colorations and transversal sizes [14,15].

This report aims to describe four cases of early-age patients treated with AMCOP elastomeric devices. This case series is illustrative of 4 cases among 38 in developmental age treated with Bio-Activators that, at the end of the treatment, required no additional fixed appliances.

## 2. Materials and Methods

The present retrospective clinical cases have been treated in the Dental Unit of the University of Bari (Italy), in full accordance with ethical principles, including the World Medical Association Declaration of Helsinki and the additional requirements of Italian law. Furthermore, the University of Bari, Italy, classified the study to be exempt from ethical review as it carries only negligible risk and involves the use of existing data that contains only non-identifiable data about human beings. The patient signed a written informed consent form.

### 2.1. AMCOP Bio-Activator Devices

#### 2.1.1. Bio-Activator for young children

**Figure 1:**
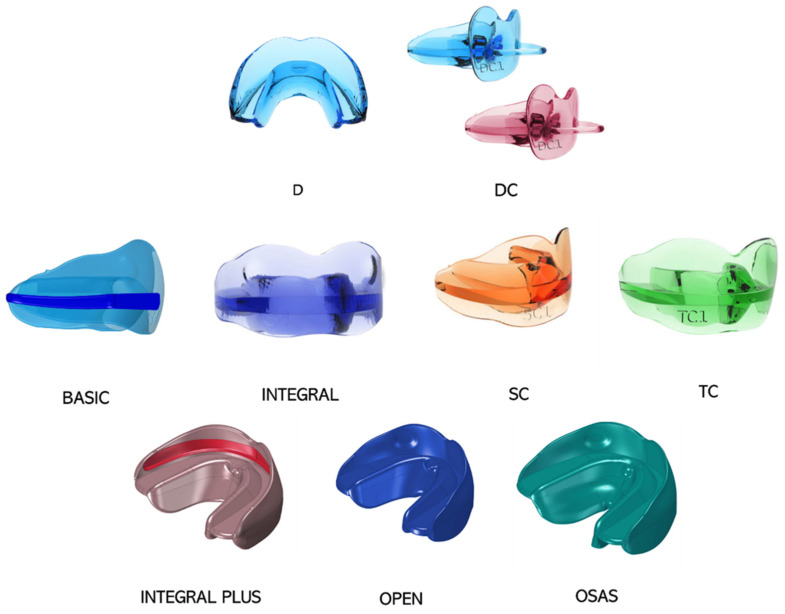
AMCOP devices available on the market.

Bio-Activators for very young children D and DC (Figure 1), respectively, without and with “pacifier” grip are used in deciduous dentition with a functional, orthopedic-osteopathic action. They are more specifically indicated for transverse deficit and open bite, due to prolonged use of the pacifier and are ideal to replace the pacifier, stimulating the correct growth of the jaws.

#### 2.1.2. First Class Bio-Activator

BASIC (Figure 1) presents an occlusal plane thicker in the anterior area to raise the vertical dimension in deep-bite patients.

First class devices are available in four different arch shapes:

− S: For mesocephalic cranial index and oval dental arches
− OS: for mesocephalic cranial index and squared dental arches
− F: For dolichocephalic cranial index
− C: For mesocephalic cranial index and squared dental arches

**Figure 2:**
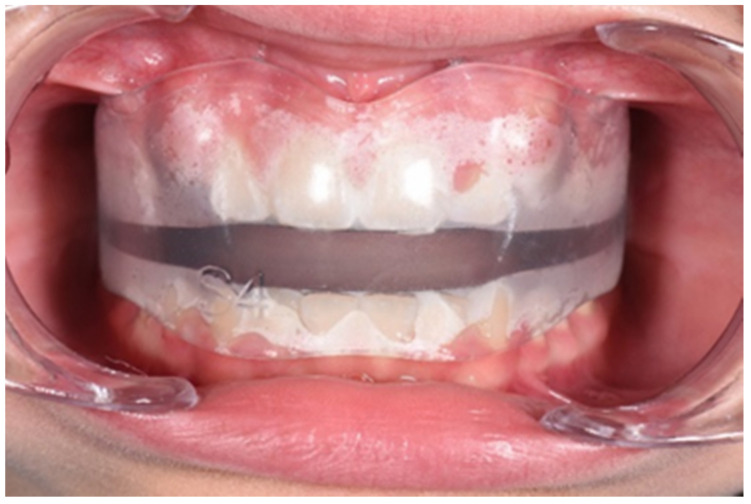
Intraoral photo of the AMCOP INTEGRAL device which presents a flat occlusal plane for class I malocclusions.

INTEGRAL (Figure 1 and Figure 2) presents a flat occlusal plane and it is indicated for a more specifically orthopedic action, correction of alterations of occlusal curves, such as anterior and lateral open bite, and a correct balance of the dental arches.

#### 2.1.3. Second Class (SC) Bio-Activator

SC (Figure 1) is equipped with a mandibular anterior sliding plane to promote the advancement of the mandibular jaw, the correction of the overjet through the retroinclination of the upper incisors, the proinclination of the lower ones and the improvement of TMJ dysfunction.

#### 2.1.4. Third Class (TC) Bio-Activator

TC (Figure 1) is indicated for the treatment of third class dysmorphosis by positioning the upper teeth in an anterior sliding plane, while it exerts a posterior pressure on the lower arch, for a braking action of mandibular growth.

#### 2.1.5. Novel Bio-Activators

− INTEGRAL PLUS (Figure 1) is equipped with an internal groove, so it can be used in conjunction with multibrackets appliances.
− OPEN (Figure 1) is characterized by a posteriorly raised occlusal plane and it is indicated for the treatment of skeletal open bite.
− ELASTO-OSAS (Figure 1) is designed for the treatment of night snoring, sleep apnea. It features a front sliding plane and an accentuated lingual shield to promote the correct positioning of the tongue on the palate [14,15].

## 3. Case Series Presentation

### 3.1. Clinical Case #1

**Figure 3:**
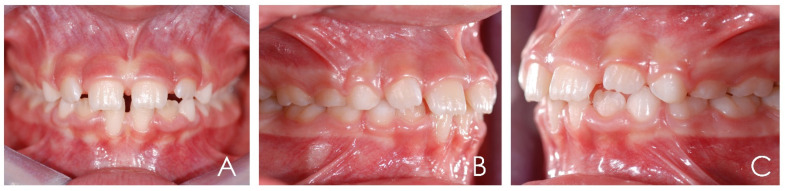
Initial intraoral photographs of the subject (10-year-old) showed canine class I, deep bite, vestibular inclination of the frontal teeth and atypical swallowing. (**A**) Frontal view; (**B**,**C**) lateral view.

**Figure 4:**
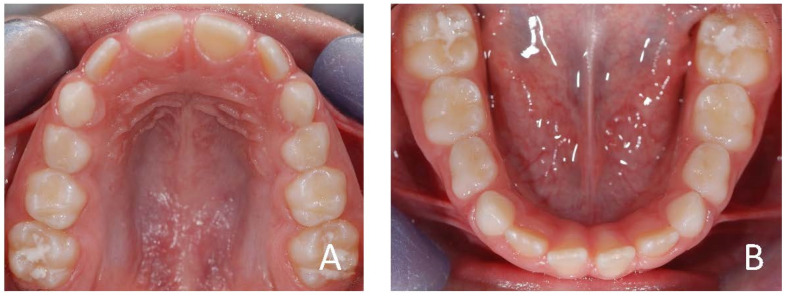
Initial occlusal photos of dental arches (10-year-old). (**A**) Upper arch; (**B**) lower arch.

**Figure 5:**
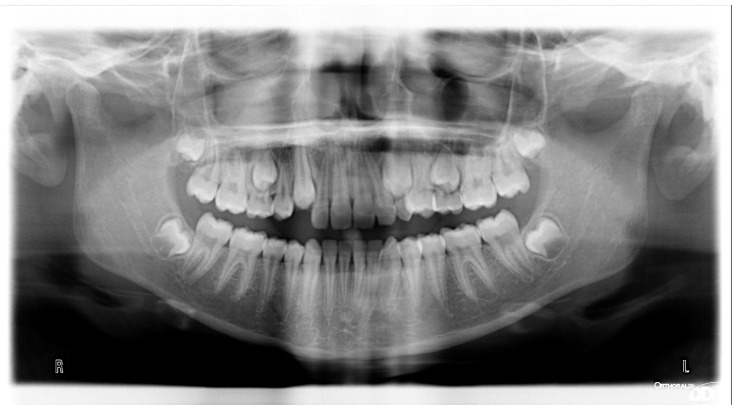
Radiographs of the subject at the screening. Orthopantomography X-ray before treatment (10-year-old).

**Figure 6:**
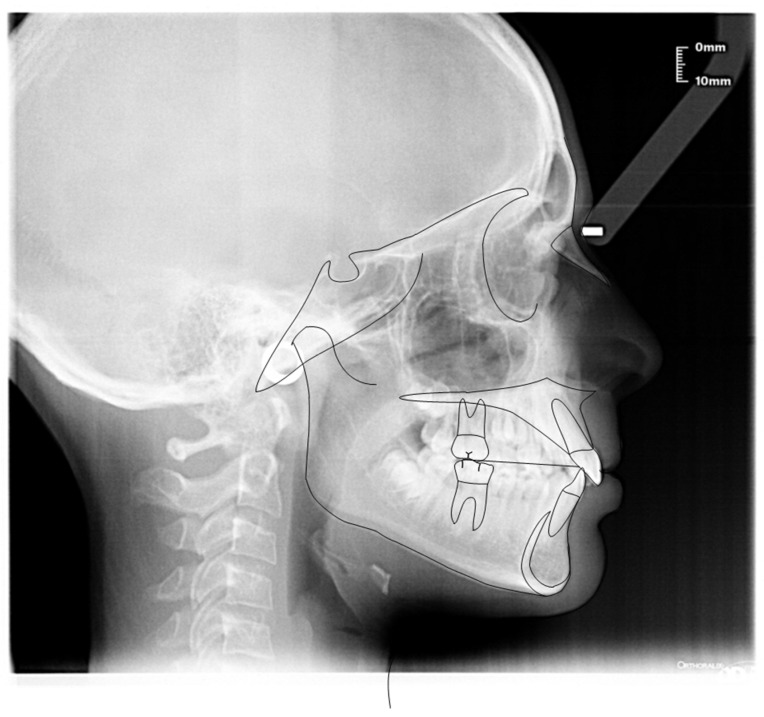
Cephalometric tracing (DeltaDent software) before treatment (10-year-old) reveals a skeletal class I.

**Figure 7:**
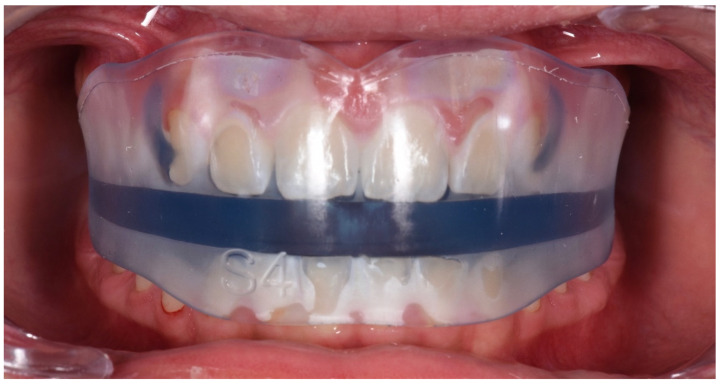
Intraoral photograph with the AMCOP Integral device.

**Figure 8:**
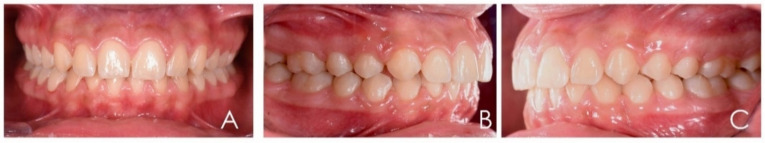
Intraoral photographs after the treatment and a 3-year follow-up period (14-year-old). (**A**) Frontal view; (**B**,**C**) lateral view.

**Figure 9:**
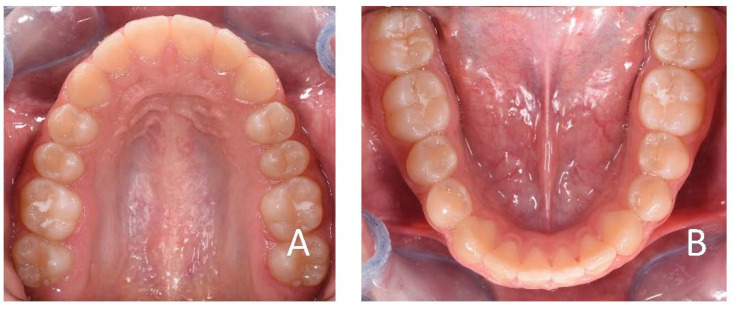
Final occlusal photos of dental arches (14-year-old). (**A**) Upper arch; (**B**) lower arch.

**Figure 10:**
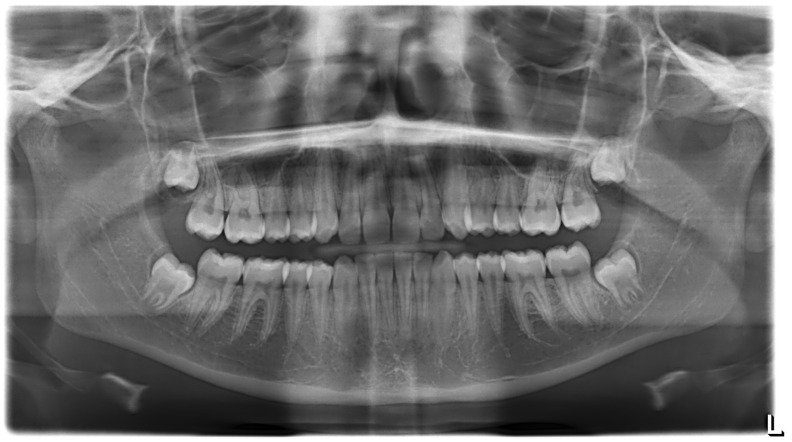
Radiographs of the subject at the end of the treatment. Orthopantomography X-ray after treatment and a 3-year follow-up period (14-year-old).

**Figure 11:**
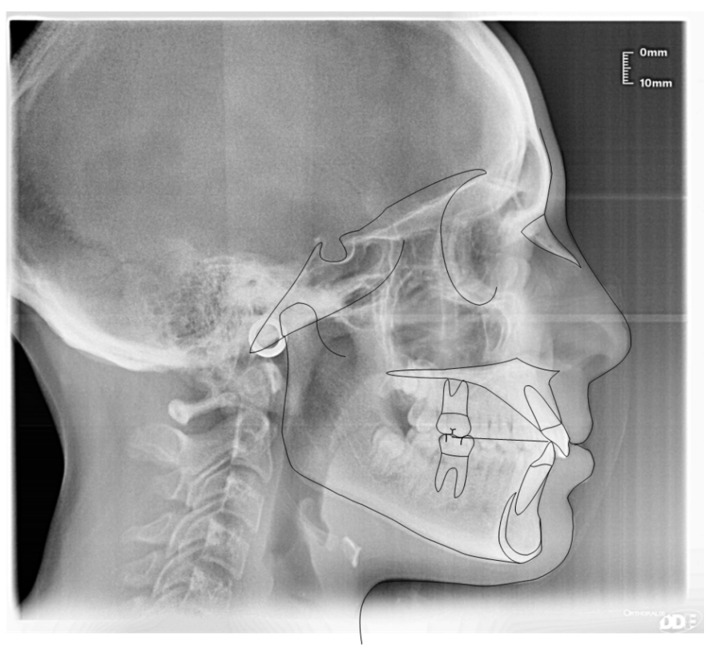
Cephalometric tracing (DeltaDent software) after treatment and a 3-year follow-up period (14-year-old).

The patient was a 10-year-old female with atypical swallowing treated with AMCOP INTEGRAL. Extraoral examination revealed a symmetrical face, competent lips and convex profile. Intraoral examination before treatment showed mixed dentition, mild molar class II on the right side and molar class I on the left side, deep bite and vestibular inclination of the frontal teeth caused by atypical swallowing. Cephalometric analysis (Deltadent^®^ Lana, Bolzano, Italy) (Outside Format, Meola Fabio. Pandino (CR), Italy) before treatment revealed a skeletal class I (SNA 82°; SNB 78.6°; ANB 3.4°; Wits 1.5 mm) (Figure 3, Figure 4, Figure 5 and Figure 6; Table 1). The proposed treatment plan made use of the INTEGRAL S device (Figure 7) and speech therapy. The patient wore the device every night and 1 hour during the day for 6 months and only overnight for another 6 months (Figure 8, Figure 9, Figure 10 and Figure 11; Table 2). The active phase was followed by a restraint phase during which the patient used the device every other night. At the end of treatment, the patient showed a dentoskeletal class I and corrected overjet and overbite; the device also allowed the tongue reeducation and atypical swallowing correction.

### 3.2. Clinical Case #2

**Figure 12:**
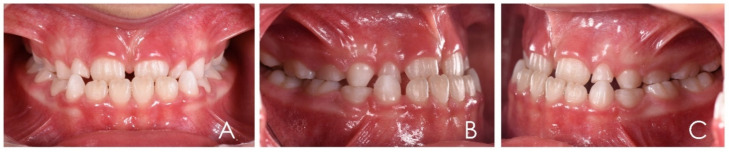
Intraoral photographs of the subject with skeletal class III malocclusion (7-year-old). (**A**) Frontal view; (**B**,**C**) lateral view.

**Figure 13:**
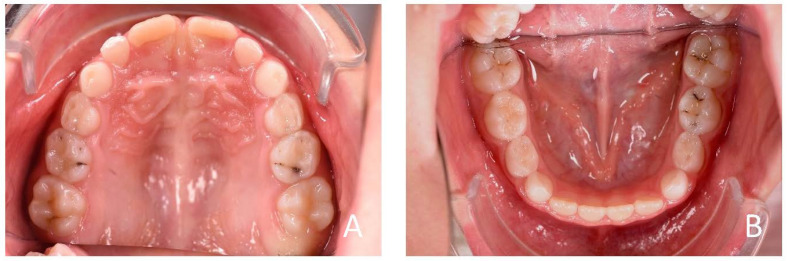
Initial occlusal photos of dental arches (7-year-old). (**A**) Upper arch; (**B**) lower arch.

**Figure 14:**
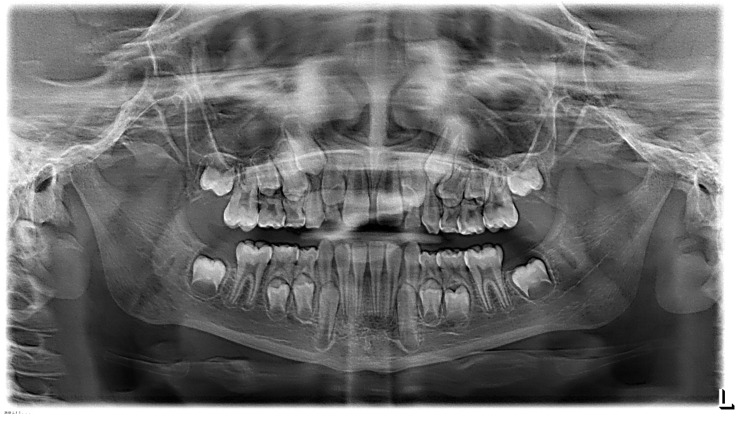
Radiographs of the patient at the screening. Orthopantomography X-ray before treatment (7-year-old).

**Figure 15:**
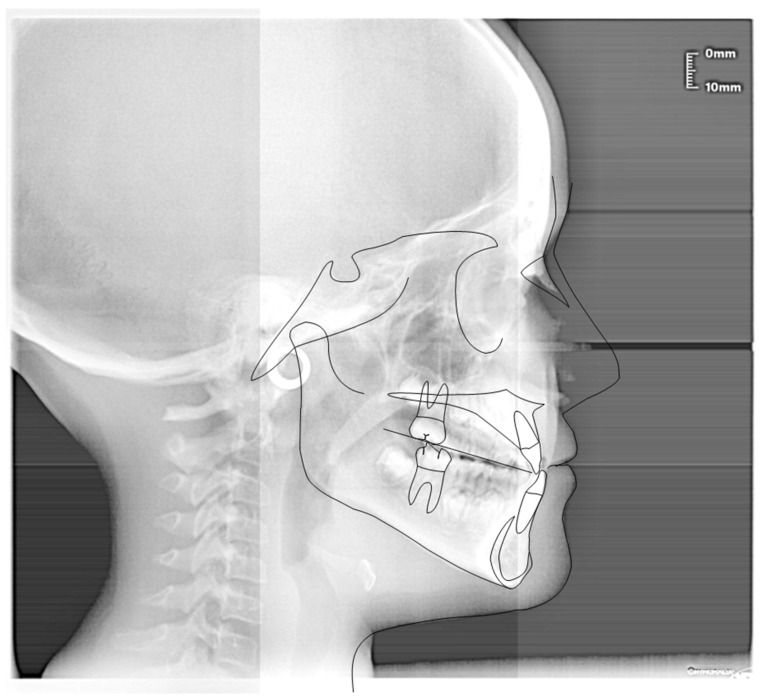
Cephalometric tracing (DeltaDent software) before treatment reveals a skeletal class III malocclusion (7-year-old).

**Figure 16:**
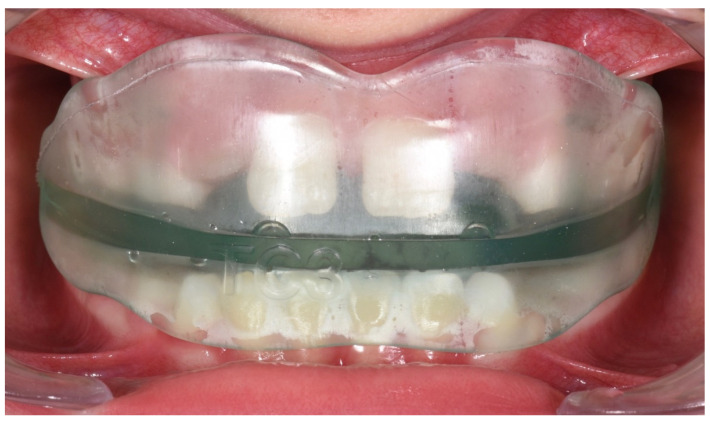
Intraoral photograph of the patient wearing the AMCOP TC.

**Figure 17:**
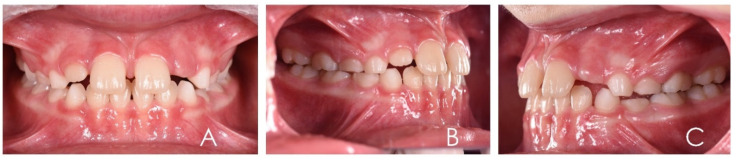
Intraoral photographs at the end of the treatment (8-year-old). (**A**) Frontal view; (**B**,**C**) lateral view.

**Figure 18:**
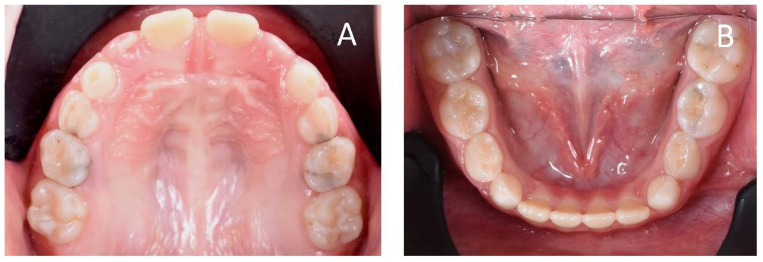
Final occlusal photos of dental arches (8-year-old). (**A**) Upper arch; (**B**) lower arch.

**Figure 19:**
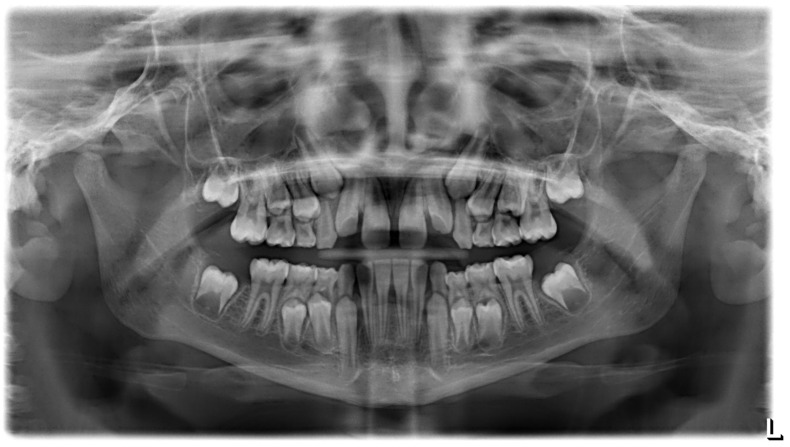
Radiographs of the subject at the end of the treatment. Orthopantomography X-ray after treatment (8-year-old).

**Figure 20:**
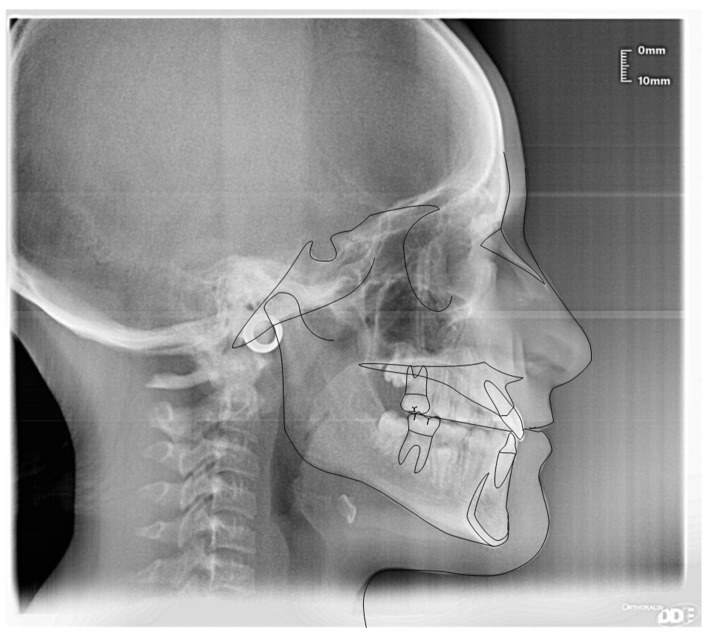
Cephalometric tracing (DeltaDent software) after treatment shows a skeletal class I (8-year-old).

The patient was a 7-year-old Caucasian female. The face was symmetric with a prognathic mandible and concave profile. Intraoral examination revealed molar and canine class III malocclusion and anterior crossbite (Figure 12, Figure 13 and Figure 14). The cephalometric analysis (Figure 15; Table 3) made with Deltadent^®^ software showed skeletal class III malocclusion (SNA 82.4°; SNB 83.2°; ANB −0.8°; Wits −8.5 mm). The device used in this case was AMCOP TC (Figure 16), indicated for the treatment of third class dysmorphism because it promotes the correct position of the upper arch in an anterior sliding plane and exerts a posterior pressure on the lower arch, curbing mandibular growth. The treatment consisted of an active phase during which the device was used every night and 1 hour during the day for 6 months and only overnight for another 6 months and a following restraint phase. After treatment, molar and canine class I relationships, proper overjet and overbite and improvement of the skeletal class were achieved. However, after this first interceptive stage, strict control of the patient occlusion and mandibular growth should be performed (Figure 17, Figure 18, Figure 19 and Figure 20, Table 4).

### 3.3. Clinical Case #3

**Figure 21:**
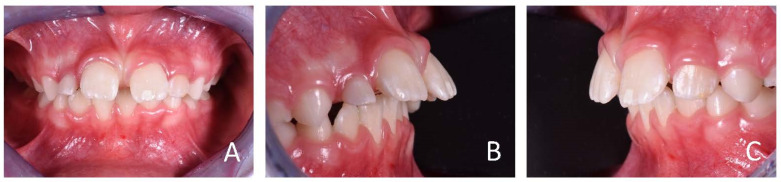
Intraoral photographs of the subject before the treatment (7-year-old). (**A**) Frontal view; (**B**,**C**) lateral view.

**Figure 22:**
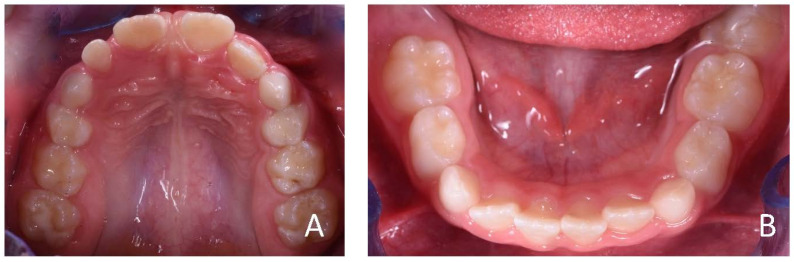
Initial occlusal photos of dental arches (7-year-old). (**A**) Upper arch; (**B**) lower arch.

**Figure 23:**
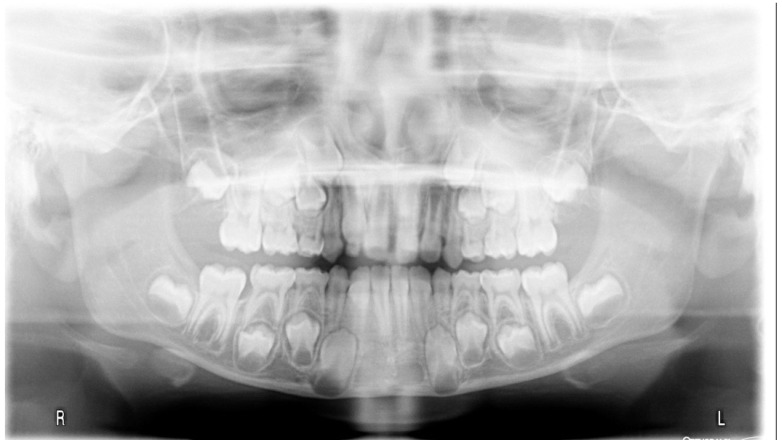
Radiographs of the patient at the screening. Orthopantomography X-ray before treatment (7-year-old).

**Figure 24:**
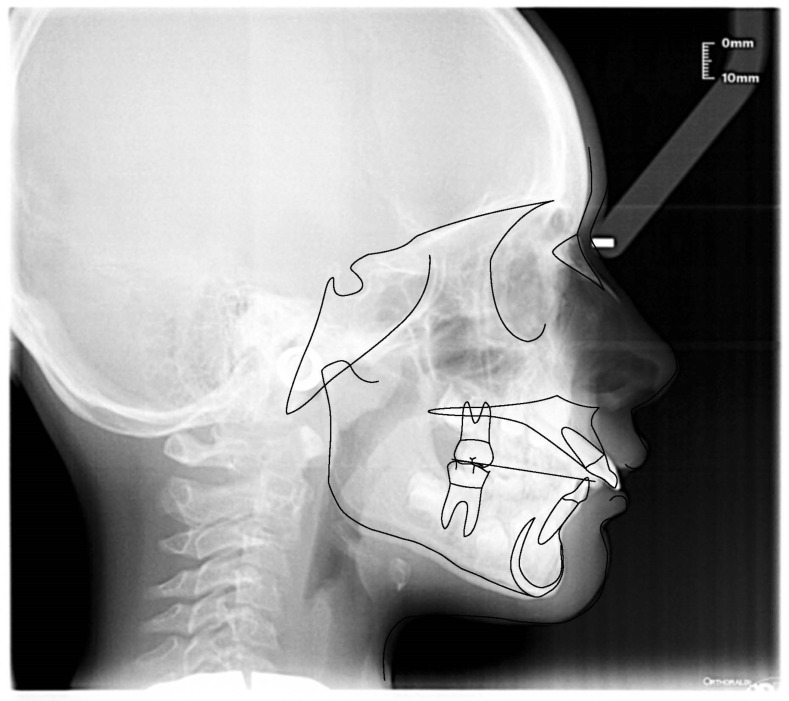
Cephalometric tracing (DeltaDent software) before treatment (7-year-old).

**Figure 25:**
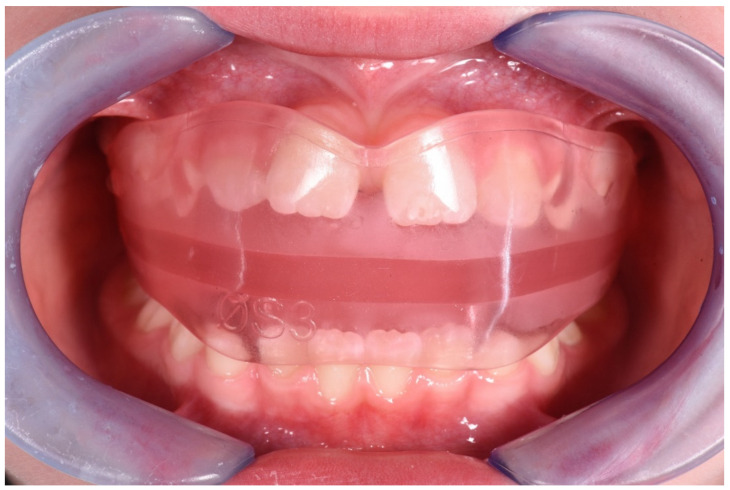
Intraoral photograph of the patient wearing the AMCOP OS.

**Figure 26:**
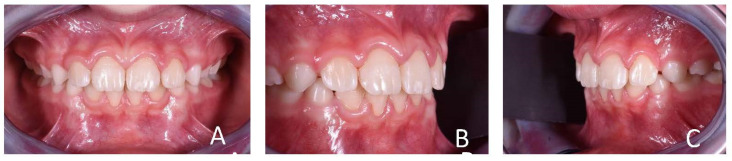
Intraoral photographs at the end of the treatment (9-year-old). (**A**) Frontal view; (**B**,**C**) lateral view.

**Figure 27:**
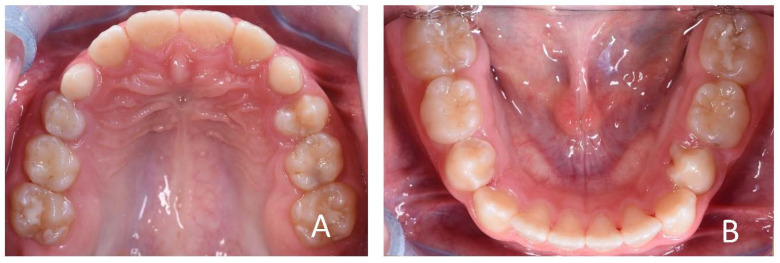
Final occlusal photos of dental arches (9-year-old). (**A**) Upper arch; (**B**) lower arch.

**Figure 28:**
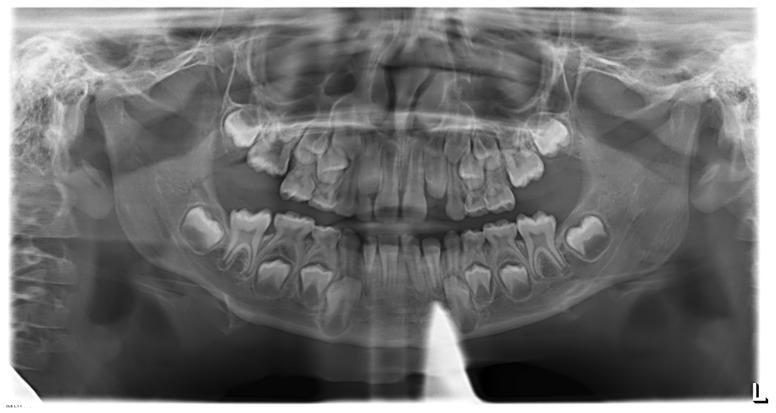
Radiographs of the patient at the screening. Orthopantomography X-ray after treatment (9-year-old).

**Figure 29:**
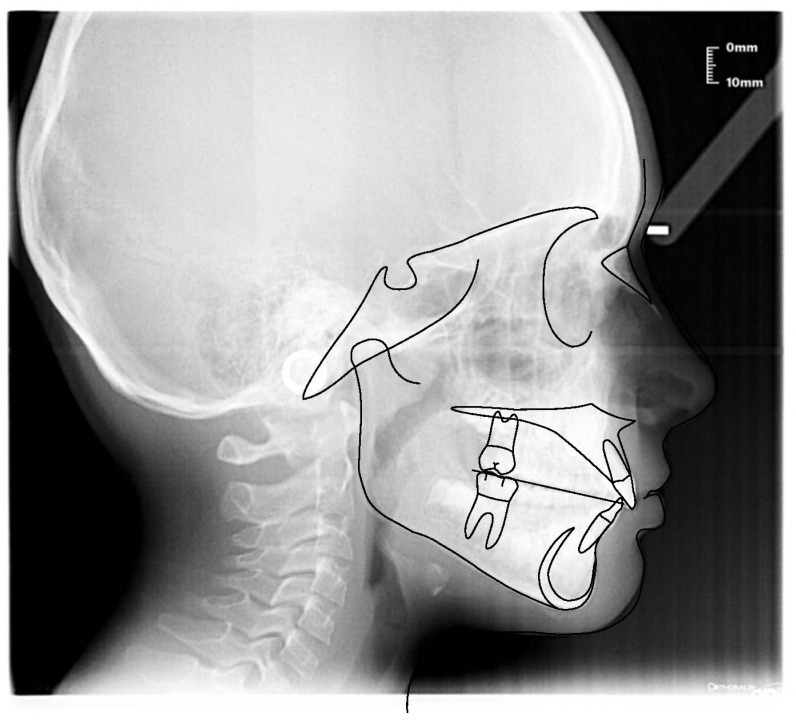
Cephalometric tracing (DeltaDent software) after treatment (9-year-old).

The patient was a 7-year-old male. The face was symmetric and the profile was convex. Intraoral photos showed deep bite, augmented overjet and a vestibular inclination of the upper incisors (Figure 21, Figure 22 and Figure 23). Cephalometric analysis (Figure 24; Table 5), performed with DeltaDent^®^ software, revealed skeletal class II malocclusion (ANB 7.7°, Wits 4.2), esoinclination of upper and lower teeth (IS^II 103°, IS^NS 119°, II^GoGn 104°). The patient was treated with AMCOP INTEGRAL OS (Figure 25) for 8 months and with AMCOP SC for 12 months. The patient was instructed to wear the appliances during the nighttime and 1 hour during the day for six months and then only during the night. At the end of treatment, correction of the skeletal class (ANB 3.2°; Wits −0.8 mm), a proper inclination of the upper incisors (IS^NS 103,4°), an improvement of the interincisal angle (IS^II 122°) and adequate overjet and overbite were obtained (Figure 26, Figure 27, Figure 28 and Figure 29; Table 6).

### 3.4. Clinical Case #4

**Figure 30:**
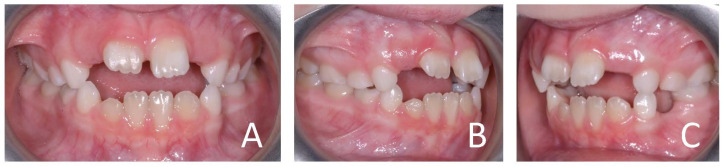
Intraoral photographs of the subject before the treatment (8-year-old). (**A**) Frontal view; (**B**,**C**) lateral view.

**Figure 31:**
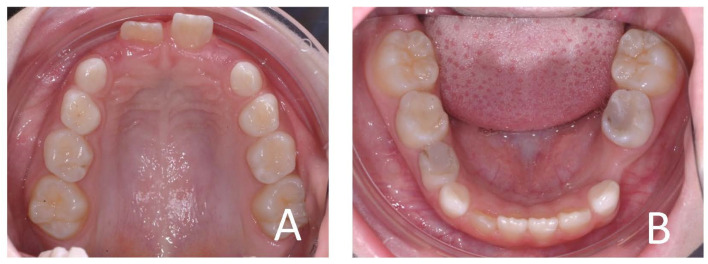
Initial occlusal photos of dental arches (8-year-old). (**A**) Upper arch; (**B**) lower arch.

**Figure 32:**
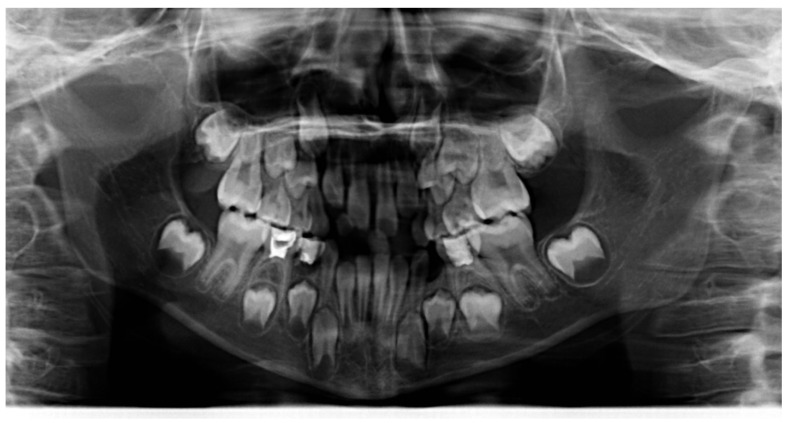
Radiographs of the patient at the screening. Orthopantomography X-ray before treatment (8-year-old).

**Figure 33:**
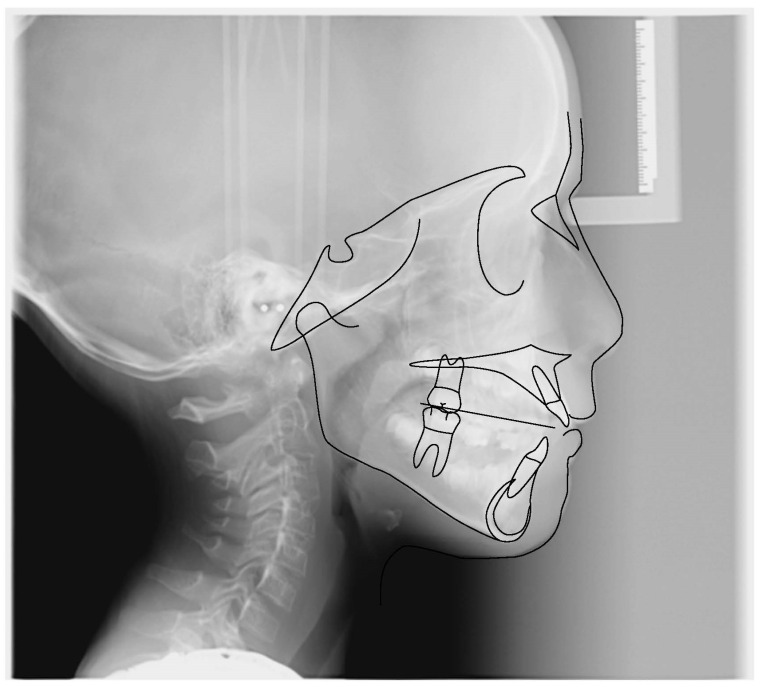
Cephalometric tracing (DeltaDent software) before treatment (8-year-old).

**Figure 34:**
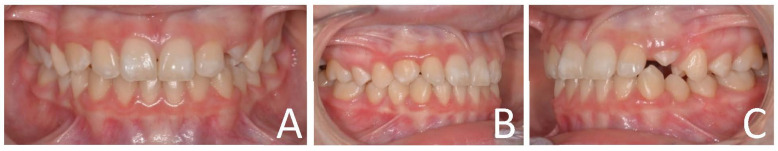
Intraoral photographs of the subject after treatment and a 3-year follow-up period (12-year-old). (**A**) Frontal view; (**B**,**C**) lateral view.

**Figure 35:**
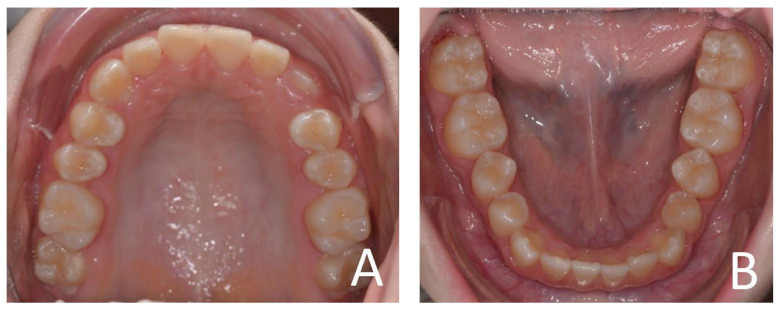
Occlusal photos of dental arches after treatment and a 3-year follow-up period (12-year-old). (**A**) Upper arch; (**B**) lower arch.

**Figure 36:**
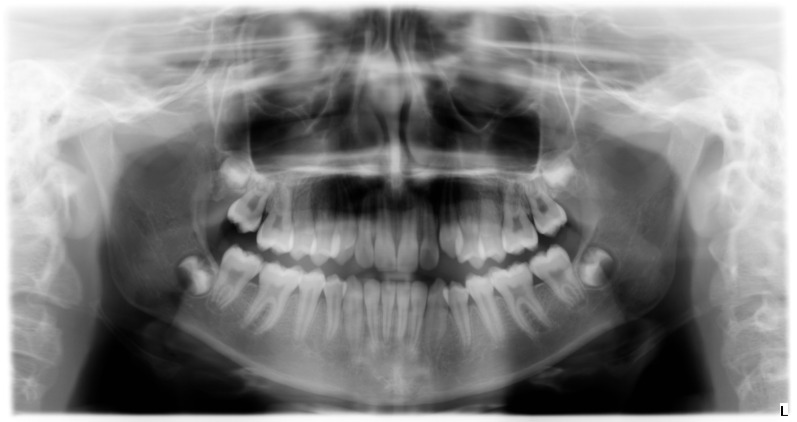
Radiographs of the patient at the screening. Orthopantomography X-ray after treatment and a 3-year follow-up period (12-year-old).

**Figure 37:**
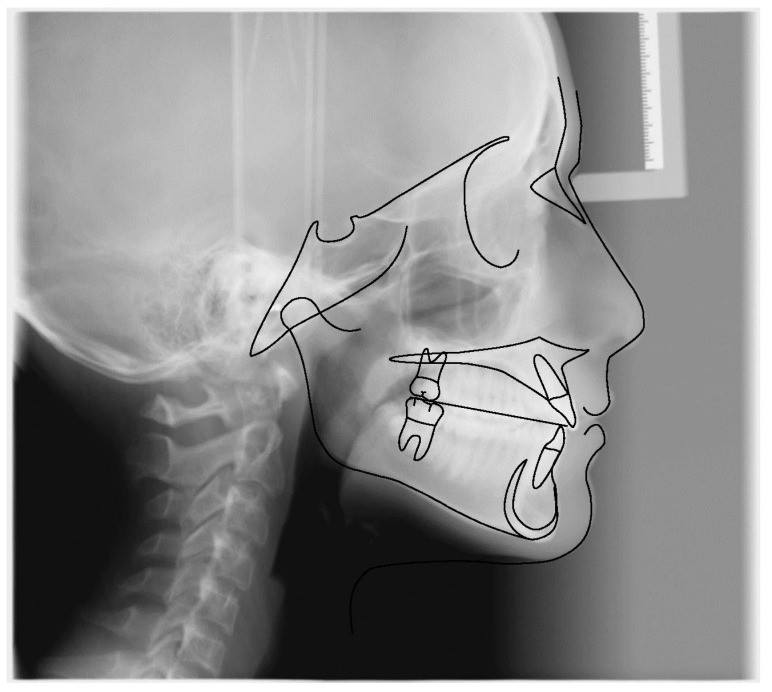
Cephalometric tracing (DeltaDent software) after treatment and a 3-year follow-up period (12-year-old).

The patient was an 8-year-old female. She had a long-face and incompetent lips. Intraoral examination showed anterior open bite, contraction of the upper arch, lack of space for the eruption of permanent teeth and atypical swallowing. Patient’s parents reported a prolonged use of the pacifier (Figure 30, Figure 31 and Figure 32). Cephalometric analysis (Figure 33; Table 7) (DeltaDent software) revealed mild skeletal class II malocclusion (SNA 74.5°; SNB 70.3°; ANB 4.2°; Wits 0.1 mm) and skeletal open bite (AnsPns^GoGn 37°; SN^GoGn 42.1°). The treatment plan included speech therapy, elastodontic therapy with AMCOP Open Bio-activator and elastodontic restraint. During the active phase, the patient wore the device during the night and 1 hour during the day for 6 months and only during the night for the other 12 months. After treatment, correction of dentoskeletal open bite (AnsPns^GoGn 23.3°; SN^GoGn 36.1°), the expansion of the upper arch, which favored a correct eruption of the permanent teeth, and the restoration of a correct function of the muscles of lips and tongue were achieved (Figure 34, Figure 35, Figure 36 and Figure 37; Table 8).

## 4. Discussion

### 4.1. Evolution of Functional Appliances: From Monobloc to Elastodontic Devices

Functional appliances are a group of active or passive appliances which act on the orofacial musculature and transmit forces to the teeth and basal bones, resulting in orthodontic and orthopedic changes [16]. In the early XX century, functional appliances were introduced in Europe and quickly gained popularity, while they were initially neglected in the United States where a more mechanical view of occlusion was widespread [17]. Functional orthodontics was introduced in the American orthodontic practice around the 1960s, especially thanks to the influence of Harvold [18]. In 1902, Pierre-Robin described the forerunner of functional appliances, a vulcanite monobloc, for the treatment of mandibular retrusion, improving the pharyngeal airway and preventing glossoptosis in patients affected by Pierre-Robin syndrome, characterized by cleft lip and palate and micromandible [19]. In 1909, Viggo Andresen developed his activator. It consisted of a plastic block covering the palate and the teeth of both arches and a lingual horseshoe flange on the mandibular teeth, to guide the mandible forward about 3 to 4 mm in occlusion and provide mandibular advancement to correct class II malocclusion. Therefore, Karl Häupl, an Austrian pathologist and periodontist, saw the potential of the Andresen appliance and became an enthusiastic advocate of what he and Andresen, called the “Norwegian system” [19,20].

In subsequent years numerous types of functional devices have been developed, usually bearing the name of their inventor such as Bimler, Balters, Fränkel, Planas, Cervera, Bass and Twin Block [21,22,23,24,25,26,27]. Traditional functional devices consisted of rigid and semi-rigid structures. The evolution of inorganic, elastic and biocompatible materials led to the realization of preformed appliances named elastomeric devices [14]. The precursor of the elastodontic appliance was the positioner, a single elastomeric device with intercuspation for the lower and upper teeth in normal occlusion. It was designed in 1945 by H.D. Kesling as a finishing appliance or as retention after a multibrackets therapy that did not require impressions or setup creation. The elastomeric material of positioners usually allows minor tooth movement after orthodontic treatment [28,29,30]. In the 1950s, Soulet and Besombes, from the French school of functionalist orthopedics, developed a functional appliance made of natural rubber and called as “activator”. The activator, thanks to its elasticity, was able to induce a well-controlled skeletal effect and to renormalize the position of the mandible and the different bones of the entire cranial system. Besombes defined this therapy as orthopedics of interception (mastic-reflex-therapy) [31,32]. Bergersen, in 1975, designed a prefabricated elastomeric appliance to correct malocclusions. It presented the combined features of a functional appliance and a positioner and was called the Occlus-O-Guide^®^ or Eruption Guidance Appliance (EGA) (ORTHO-TAIN, Inc, Bayamon Gardens, PR). The characteristics shared with functional devices were the mandibular advancement in order to correct Class II sagittal discrepancies and a vertical opening in the anterior region to provide a greater vertical development of the posterior teeth [33,34,35]. Thanks to its soft material, elastodontic appliances do not cause damage to oral soft and hard tissues during use [36].

Numerous types of elastodontic devices are available on the market:

- EF devices designed by Daniel Rollet (Éducation Fonctionnelle, Orthoplus^®^, Igny, France);
- T4K and POT (Trainer For Kids e Pre-Orthodontic Trainer), actually called Myobrace^®^ (MRC Myofunctional Research, Helensvale, Australia);
- LM-Activator (LM, Parainen, Finland);
- MFS (Multi-Function System) by Duran von Arx (Orthodontic World, Barcelona Spain);
- Muppy^®^ Oral Screen developed by Hintz (DHD, Herne, Germany)
- Occlus-o-Guide^®^, Nite-Guide^®^, Class III^®^ and Healthy Start™ (Sweden and Martina)
- AMCOP^®^ (*Armonizzatori Multifunzionali Cranio-Occluso-Posturali*, Cranio-Occlusion-Postural Multifunctional Harmonizers) by Micerium [37].

### 4.2. Structural Features of Elastodontic Devices

Different elastomeric appliances have been designed to treat many forms of malocclusions [38]. Generally, these devices feature two double-matched planes, upper and lower, and two flanges, one on the vestibular side and one on the lingual side. Teeth are positioned in a central area that can be with indentations, as a positioner, or with a free central space, avoiding teeth constriction or the generation of orthodontic movement [39]. The upper and lower planes can be collocated in a different position to promote or stop mandibular advancement, as a functional appliance [40]. The occlusal plane can be flat, thicker in the anterior region or in the posterior one, to control verticality and promote, or not, the eruption of the posterior teeth. The adjunctive ramp on the lingual flange provides to guide the tongue on the palate and is determinant for functional rehabilitation in cases of atypical swelling. The employed materials are a soft silicone elastomer or polymer/elastomer combination to produce light and biological elastic forces without traumatizing the oral mucosa [14].

### 4.3. Indication of Elastodontic Devices

The purpose of the elastodontic devices as well as all the functional devices is to work and stimulate groups of muscles that influence the positions of the mandible, tongue and dental arches as a consequence, with light and biological forces [41]. The scientific literature evidences the resolution or improvement of the basal second class with augmented overjet and deep bite malocclusion since they stimulate mandibular growth and protrusion, retrusion of the upper incisors, protrusion of the lower incisors, improvement of molar relationship [12,29,42,43]. Such devices can be used in association with fixed orthodontic appliances in every age since it shows a reduction of treatment times and contributes to creating an ideal arch form [44]. Another interesting use of elastodontic devices is the finishing of the dental occlusion in the final phase of orthodontic treatment. They consent to the operator to conclude this delicate phase of treatment calmly and smartly by using a customized device that is used only at night, acting on every single tooth, improving the occlusal intercuspation, and favoring a normal functional reeducation [45]. These devices seem to be an interesting tool for the treatment of temporo-mandibular-joint (TMJ) disorders but further studies are needed to prove this aspect [46]. The studies suggested that orthodontic treatment with elastodontic appliances can be successful in reducing the intensity of signs and symptoms of obstructive sleep apnea syndrome (OSAS) in preschool children [47,48]. Elastodontic devices are better performing in deciduous or mixed dentition, since we can find an improvement of sagittal and vertical relations and a spontaneous alignment of incisor position [39,40]. They also allow preventing malocclusion such as crowding, spacing, rotations, overjet, overbite, second class molar relationship and temporomandibular dysfunction [34]. However, elastodontic devices can be used in every age in accordance with the treatment needed (e.g., adults finishing) or as support appliance after fixed orthodontic treatment [44].

### 4.4. Orthopedic-Functional Orthodontic Therapy with Elastomeric Devices

Elastomeric devices can treat malocclusions, correct teeth positions and influence growth using delicate elastic forces. Developmental age (deciduous or mixed dentition) is the main period in which these devices are adopted to correct orthopedic and/or orthodontic anomalies [11,14]. The treatment plan is defined by the patient developmental stage and adolescent growth spurt. For these reasons, it is important to evaluate the individual skeletal maturity. Different indicators have been used to this aim: chronological age of the patient, cervical vertebral maturation (CVM), skeletal maturation of hand-wrist and increase in height. Chronological age is the easiest method with good reliability as a predictor for the adolescent growth spurt. The hand-wrist stages are the best indicator for the peak velocity stage of skeletal maturation, albeit, together with the CVM, they offer no benefit to chronologic age in evaluating or foreseeing facial growth timing. Stature could be a more useful predictor than age because it can be measured frequently without X-Rays exposition [49,50]. In another work, the authors concluded that cervical vertebral stages of maturation (6 stages) are related to mandibular growth changes during puberty and showed that cervical vertebrae radiography is comparable to the hand-wrist for skeletal age assessment [51]. Baccetti, Franchi and McNamara proposed an improved method of the CVM to evaluate the peak in mandibular growth using only one cephalogram focusing only on the body morphology of the second to fourth vertebras. Their body is visible even if they were protected with a radiation collar [52]. Therefore, five cervical vertebral maturational stages CVMS have been proposed and the mandibular growth peak occurred between the second and the third stage [52]. Elastodontic appliances are adopted for different clinical purposes: preventive, interceptive and retention.

### 4.5. Preventive Phase

The preventive phase aims to eliminate external etiological factors disadvantageous for patient growth. This is the fundamental objective of orofacial orthopedics (a term derived from Greek with the meaning of “proper education”) [23]. With interceptive orthodontics, these two phases are included in the definition of early orthodontic treatment (EOT) [53]. According to Ackerman and Proffit [54], preventive orthodontics is “prevention of potential interferences with occlusal development” while interceptive orthodontics is “elimination of existing interferences…[omissis]…involved in the development of the dentition”. However, most of the time there is not a clear demarcation line between a potential or an existing interference. It is known that growth variations of all skeletal units depend on their functional matrices. These changes concern bones shape, size and spatial position [55]. Thus, correcting altered functions or oral bad habits is possible to restore a proper growth pattern of jaws and the relationship of oral structures (e.g., teeth) [45]. Before moving on to the next paragraph, it is important to summarize the indications for early orthodontic intervention [56]:

− Anterior and posterior crossbites;
− Ankylosis of teeth;
− Augmented protrusions and diastemas (linked to injury or traumatic avulsions);
− Anterior and lateral open bites (often in association with bad tongue or digit habits);
− Ectopic molars;
− Serious arch length differences;
− Cleft palate;
− Pseudo Class III;
− Class III malocclusion due to maxillary retrusions.

### 4.6. Interceptive Phase

Many studies have demonstrated the importance of the use of functional devices in growth subjects, demonstrating the importance of the interceptive phase [12,57,58,59]. Interceptive therapy acts on abnormal behavior of the musculature, resolving issues related to the presence of non-physiological functional spaces through the use of removable appliances [60]. In a 2011 study, authors evaluated the effect of interceptive therapy for Class II malocclusion with two different appliances, Clark’s twin block and Bergensen’s Occlus-O-guide. The results showed that both appliances were able to promote significant and obvious clinical effects. The difference between the two appliances was that the Occlus-O-guide, despite Twin Block, requires a one-step therapy, solving simultaneously skeletal, dentoalveolar and dental problems [60]. A precedent work showed that the prefabricate appliances were capable to correct many factors of the developing occlusion including overjet, overbite, open bite, spatial deficiencies and Class II molar relationship [57]. Other authors suggested that the key effect of a functional appliance is to displace the mandible forward and let the condyle grow into the fossae without producing dentoalveolar compensation [61]. In a 2021 work, authors assessed the potential effectiveness of interceptive orthodontic treatment with an elastodontic appliance in subjects with specific early signs of malocclusion (mixed dentition stage). According to findings, all subjects treated with elastodontic appliance (EA), showed a significant improvement of the overjet, overbite, crowding, and sagittal molar relationship. Conversely, as evident in the second case report, a possible effect of the treatment could be represented by the protrusion of the upper incisors while the influence of further growth should be considered in this phase. On contrary, a positive overjet facilitates a better development of the maxilla evidencing a successful interceptive treatment. Both skeletal and dentoalveolar changes have contributed to the resolution of the malocclusion, suggesting that elastodontic appliances may represent a comprehensive early treatment method [14]. These findings may support previous evidence showing that one phase of treatment with elastodontic devices followed by a long retention period, including adolescence, might be an effective and alternative approach to the conventional biphasic protocol with a functional device and further treatment with a fixed appliance [62].

### 4.7. Retention Phase

Elastodontic appliances are useful for the retention phase after an active elastodontic therapy [12]. In this study patients in mixed dentition were examined, with skeletal Class II malocclusion and the active phase was estimated at twelve months. After this period, an elastodontic appliance was used for the retention phase with this protocol: the first twelve months Occlus-o-Guide every night. It was then checked every three months and the device was worn every night for another year. Proper retention of the early preventive overbite correction seems to be dependent on two different factors: collagenous fiber formation, intraseptal fibers particularly and alveolar and vertical jaw growth. The elastodontic appliance can be used in a two-phase approach. In the first phase, an elastodontic appliance for the treatment of anterior crowding, overjet and overbite, coordination of arches and for a normal occlusion was used. In the second phase, after the extraction of bicuspid teeth, the fixed appliance was placed to close residual space and improve teeth angulations [44].

### 4.8. Comparison between Elastodontic Appliances and Traditional Functional Devices

The functional philosophy is based on the correlation between form and function: functional stimuli influence growth and dental eruption by interfering with neuromuscular mechanisms [16]. Passive activators act “drop” only through functional stimuli, while active ones exert a mechanical action by adding components (springs, screws, extraoral tractions). Classification of appliances is reported in Table 9 [63].

Elastodontics [19] alongside the typical mechanisms of traditional braces (springs, screws, myofunctional) employs elastic forces exerted as a result of the total incorporation of the teeth by means of gums and polymers. These passive devices represent a synthesis of traditional functional devices using the same principles: they have a vestibular and a lingual flange that leave a free central area. The vestibular flanges recall the function of the classic Lip-Bumper and vestibular shields, eliminating the pressures of the perioral muscles. The lingual ramp, together with the button, guides the tongue towards the palatine spot like the palatine resin button of the Cervera-Bracco plates. The mandibular sliding plane of the AMCOP SC for the treatment of the second skeletal classes recalls the bite blocks of Clark’s Twin block for mandibular advancement. Elastodontic devices have different advantages compared to traditional functional devices:

- Simple patient compliance as they need to be worn fewer hours during the day;
- Less invasiveness;
- Lower costs because it is often unnecessary to adopt more devices during therapy;
- Three-dimensionality;
- No construction bite is needed, so it allows you to avoid the imprint maneuver that is not very tolerated.
- These devices have some limitations regarding:
- Severe skeletal dysmorphisms;
- Rotation of the canines, premolars and molars;
- Impacted elements;
- Severe periodontal disease;
- Mobility of teeth.

The main problem with removable functional appliances is compliance [64]. These are often difficult appliances to wear as they can affect speech and oral function and, therefore, not all patients tolerate them. Failure rates of up to 34% have been reported for Twin Blocks from prospective studies [65]. Is this primarily due to non-compliance? Fixed functional appliances theoretically eliminate the problem of cooperation, but are more prone to breakage and are more expensive [66,67,68,69]. A 2021 article reported a high level of compliance with the AMCOP device, despite augmented salivation [14]. One of the most frequently encountered effects is the decrease in resting hypertonia of the mental muscle and an increase in orbicular muscle activity, usually hypotonic in cases of labial incompetence [70]. Current results indicated that treatment with the Pre-Orthodontic Trainer device showed a positive influence on the chewing and perioral musculature [71]. The muscular activity of the masseter and the anterior temporal also changes thanks to the use, for at least six months, of elastic preforms [72]. It should be remembered that the results obtained with elastomeric preforms agree with those obtained with other functional equipment, such as Fränkel devices, activators associated with extraoral traction or Twin-blocks [73]. The obtained results suggest that, thanks to the use of elastomeric equipment, it is possible to achieve the perioral muscles rebalancing similar to what can be realized with rigid functional equipment, such as the Fränkel device [74]. In a recent study, the effects of an elastomeric preform were compared to the Fränkel device: the results obtained with the two devices were comparable, even if the time required to achieve similar results was higher in the case of soft preforms. The authors conclude that elastomeric devices may represent a valid alternative to the use of rigid functional appliances [75]. Some studies have instead examined the effects of elastomeric equipment through head cephalometric analysis [76]. The main effect reported was an increase in mandibular length assessed through the Condylion-Gnathion distance and advancement of the chin assessed through the distance between the Pogonion and N-perpendicular points. The Condylion point (Co) is the most posterior and superior point of the head of the mandibular condyle, while the Gnathion point (Gn) is the meeting point between the facial plane (Nasion-Pogonion plane) and the tangent plane of the mandibular edge passing through Menton (Me). Eruption guidance appliance did not significantly alter the anterior mandibular height, like other functional appliances [68]. The eruption guidance appliance presented a similar effect to other functional appliances, causing lingual tipping, linear retrusion and inhibition of the vertical development of the upper incisors. This is because the Occlus-O-Guide is thicker in the anterior region and inhibits the extrusion of the incisors, but it does not establish a contact with posterior upper teeth, so promotes the vertical development of superior molars [76]. Occlusal changes in 167 treated children in the mixed dentition stage, with crowding, overjet of 3 mm and lack of tooth-to-tooth contact between the incisors, overbite of 3 mm and lack of tooth-to-tooth contact between the incisors after treatment with Occlus-O-Guide recorded in another work [57]. Treatment began when the first primary incisors exfoliated and ended when all permanent incisors and first molars fully erupted. He found that overjet decreased from 3.1 to 1.9 mm and overbite from 3.2 to 2.1 mm. Moreover, a good alignment of the incisors was observed in 98% of the treated children. This alignment was possible because the eruption guidance appliance was designed to solve crowding by expanding the dental arches [35,36,37,38,39,40,41,42,43,44,45,46,47,48,49,50,51,52,53,54,55,56,57,58,59,60,61,62,63,64,65,66,67,68,69,70,71,72,73,74,75,76,77]. It was concluded that the Fränkel group, the EGA group and the EGA subgroup had significantly greater resorption than the control group. There was no difference in the amount of resorption between the Fränkel and the EGA groups [78]. It should be remembered that in all existing works the elastomeric preforms were used only for the treatment of skeletal and/or dental Class I and II malocclusions, excluding Class III cases a priori. To date, no scientific papers are available that evaluate the effectiveness of elastomeric devices in class III treatment [79].

### 4.9. Comparison between Bio-Activators and Other Elastodontic Devices

AMCOP Bio-activators (Figure 1) represent an evolution of the elastodontic appliances as they simultaneously harmonize maxillary and mandibular skeletal bases and are ideal for levelling inclined, rotated and twisted occlusal planes to obtain a correct function and a good balance of the masticatory system. The rehabilitative action of the Bio-Activator is reflected on the entire stomatognathic system: teeth, alveolar bones, masticatory muscles, TMJ, cheeks, lips, tongue, soft tissues, salivary glands, mandibular and maxillary bones, innervation and vascularization and, therefore, on the cervico-postural system [14,80]. Bio-activators have some features that differentiate them from other elastodontic devices available on the market. The availability of a wide range of devices gives the possibility to choose between four different arch shapes according to the clinical case and to treat different types of malocclusions thanks to different occlusal planes. The thermoactive and superelastic material allows one to apply changes during therapy with appropriate instruments and reduce the incidence of breakages. The Bio-Activators are equipped with thin and high shields and a lingual ramp on all forms that allows to reeducate tongue posture and function [14,15]. They are able to create an “elastodontic space” which represents the ideal space between tongue and lips where teeth are allowed to move. In fact, all AMCOP elastodontic appliances have no indentations because they do not act on the dental position, but they allow the correct neuromuscular balance between the muscles of the lips, tongue, elevators and depressors of the mandible, so the dental alignment will be the result of the function and not of the movement induced by the device itself. This allows obtaining a stable and long-lasting result. These devices are also effective for various muscle-tension problems and are suitable for the rehabilitation of the TMJ [81,82,83]. They are not able to correct teeth rotation and severe skeletal dysmorphosis [14,15].

The evidence of the present study reported that the subjects treated through AMCOP devices showed a clinical increase of the skeletal and dentoalveolar relationship with a consistent improvement of the malocclusion, suggesting that elastodontic appliances could represent an effective procedure with a reduced period required for the treatment. The limits of the study are correlated by the absence of a long-term follow-up for all cases. For clinical #1 and #4 cases, photos of three years follow up have been reported. Today, the lack of a sufficient quantity of randomized clinical trials in literature is the main reason for the non-applicability of a systematic approach and meta-analysis statistics in the present paper, in line with the novelty of the presented topic. More clinical studies with a sufficient follow-up will be useful to confirm the promising findings and the long-term effectiveness of the proposed interceptive approach.

## 5. Conclusions

In the four reported cases the elastodontic therapy turned out to be effective to achieve therapeutic targets with a low impact on compliance. The Bio-activators are valid aids for early treatments, reconditioning the natural growth forces of neuro-musculo-skeletal system to correct malocclusions. In addition, thermoplastic material makes these Bio-activators extremely elastic, comfortable and suitable for any dental arch conformation.

From the analysis of literature, it emerges a lack of sources about elastodontics and about AMCOP devices specifically. Thus, more research on this topic is needed.

## 6. Patents

Invention patents:

− Title: dispositivo ortodontico-elastico-armonizzatore dento cranio-facciale, scope: Italian, granted under n° 102015000057082
− Title: dispositivo ortodontico-elastico-armonizzatore dento cranio-facciale, scope: International, n° WO 2017/056010

## Figures and Tables

**Table 1:** Cephalometric analysis before treatment.

Cephalometric Analysis before Treatment	Val	Dev	Min	Med	Max	Diff
SNA	82°	N	80°	82°	84°	0°
SNB	78.6°	N	78°	80°	82°	0°
ANB	3.4°	N	0°	2°	4°	0°
sna-snp^Go-Gn	22.3°	N	15°	20°	25°	0°
S-N^sna-snp	8.9°	N	7°	10°	13°	0°
S-N^PO	14.2°	N	11°	14°	17°	0°
S-N^Go-Gn	31.2°	N	27°	32°	37°	0°
SNBa	136.6°	x	124°	129°	134°	2.6°
SND	75.1°	N	74°	76°	78°	0°
IS^II	128.9°	N	125°	130°	135°	0°
IS:N-A	3.5	N	3	4	5	0
II:N-B	3.7	N	3	4	5	0
II:A-Pog	1.7	N	−1	1	3	0
Ls:Line S	−2.1	−xx	−1	0	1	1.1
Li: Line S	0.4	N	−1	0	1	0
Cvm:S-Gn	−3.4	−xxx	−1	0	1	2.4
Mol Sup^P. Occl	99.5°	xxxx	88°	90°	92°	7.5°
N-S-Cop	135.2°	xx	117°	122°	127°	8.2°
S-Cop-Go	137.3°	N	137°	143°	149°	0°
Cop-Go-Gn	118.7°	N	115°	120°	125°	0°
Cop-Go-N	51.2°	N	48°	50°	52°	0°
N-Go-Gn	67.6°	−x	68°	70°	72°	0.4°
II^Go-Gn	94.1°	x	92°	93°	94°	0.1°
SOr:sna	62.9		0	0	0	62.9
sna:Me	63.7		0	0	0	63.7
S:N	69.6	−xx	75	78	81	5.4
snp:A	54.7		0	0	0	54.7
Go:Me	75.4	N	73.7	78.7	83.7	0
Wits	1.5	N	−2	0	2	0
IS^N-S	105.9°	x	101°	103°	105°	0.9°
Pog:N-B	0.9		0	0	0	0.9
Pog:N-B—II:N-B	−2.8	-	0	0	0	2.8

**Table 2:** Cephalometric analysis after treatment and a 3-year follow-up period.

Cephalometric Analysis after Treatment	Val	Dev	Min	Med	Max	Diff
SNA	81.8°	N	80°	82°	84°	0°
SNB	78.9°	N	78°	80°	82°	0°
ANB	2.9°	N	0°	2°	4°	0°
sna-snp^Go-Gn	22.4°	N	15°	20°	25°	0°
S-N^sna-snp	9.1°	N	7°	10°	13°	0°
S-N^PO	12.4°	N	11°	14°	17°	0°
S-N^Go-Gn	31.5°	N	27°	32°	37°	0°
SNBa	139.7°	xx	124°	129°	134°	5.7°
SND	76.1°	N	74°	76°	78°	0°
IS^II	125.1°	N	125°	130°	135°	0°
IS:N-A	3.8	N	3	4	5	0
II:N-B	4.4	N	3	4	5	0
II:A-Pog	2.2	N	−1	1	3	0
Ls:Line S	−3	−xxx	−1	0	1	2
Li: Line S	1.2	x	−1	0	1	0.2
Cvm:S-Gn	−4.7	−xxxx	−1	0	1	3.7
Mol Sup^P. Occl	95.2°	xx	88°	90°	92°	3.2°
N-S-Cop	132.3°	xx	117°	122°	127°	5.3°
S-Cop-Go	143.9°	N	137°	143°	149°	0°
Cop-Go-Gn	115.3°	N	115°	120°	125°	0°
Cop-Go-N	49.4°	N	48°	50°	52°	0°
N-Go-Gn	66°	−xx	68°	70°	72°	2°
II^Go-Gn	93.4°	N	92°	93°	94°	0°
SOr:sna	72.3		0	0	0	72.3
sna:Me	67.3		0	0	0	67.3
S:N	72.9	−x	75	78	81	2.1
snp:A	56.1		0	0	0	56.1
Go:Me	86.4	x	73.7	78.7	83.7	2.7
Wits	2	N	−2	0	2	0
IS^N-S	109.9°	xxx	101°	103°	105°	4.9°
Pog:N-B	1.5		0	0	0	1.5
Pog:N-B—II:N-B	−2.9	-	0	0	0	2.9

**Table 3:** Cephalometric analysis before treatment.

Cephalometric Analysis before Treatment	Val	Dev	Min	Med	Max	Diff
SNA	82.4°	N	80°	82°	84°	0°
SNB	83.2°	x	78°	80°	82°	1.2°
ANB	−0.8°	−x	0°	2°	4°	0.8°
sna-snp^Go-Gn	22.4°	N	15°	20°	25°	0°
S-N^sna-snp	7.6°	N	7°	10°	13°	0°
S-N^PO	20.4°	xx	11°	14°	17°	3.4°
S-N^Go-Gn	30°	N	27°	32°	37°	0°
SNBa	134.5°	x	124°	129°	134°	0.5°
SND	78.8°	x	74°	76°	78°	0.8°
IS^II	142.5°	xx	125°	130°	135°	7.5°
IS:N-A	2.2	−x	3	4	5	0.8
II:N-B	3.2	N	3	4	5	0
II:A-Pog	2.9	N	−1	1	3	0
Ls:Line S	−1.6	−x	−1	0	1	0.6
Li: Line S	−1.4	−x	−1	0	1	0.4
Cvm:S-Gn	1	N	−1	0	1	0
Mol Sup^P. Occl	106°	xxx	88°	90°	92°	14°
N-S-Cop	134.1°	xx	117°	122°	127°	7.1°
S-Cop-Go	132.8°	−x	137°	143°	149°	4.2°
Cop-Go-Gn	123.1°	N	115°	120°	125°	0°
Cop-Go-N	53.7°	x	48°	50°	52°	1.7°
N-Go-Gn	69.3°	N	68°	70°	72°	0°
II^Go-Gn	84.1°	−xxx	92°	93°	94°	7.9°
SOr:sna	45		0	0	0	45
sna:Me	51.2		0	0	0	51.2
S:N	53.7	−xxxxxx	68.7	71.7	74.7	15
snp:A	40		0	0	0	40
Go:Me	63.4	−x	66.7	71.7	76.7	3.3
Wits	−8.5	−xxxx	−2	0	2	6.5
IS^N-S	103.4°	x	101°	103°	105°	0°
Pog:N-B	1.1		0	0	0	1.1
Pog:N-B—II:N-B	−2.1	-	0	0	0	2.1

**Table 4:** Cephalometric analysis after treatment.

Cephalometric Analysis after Treatment	Val	Dev	Min	Med	Max	Diff
SNA	84.6°	x	80°	82°	84°	0.6°
SNB	84.2°	xx	78°	80°	82°	2.2°
ANB	0.4°	N	0°	2°	4°	0°
sna-snp^Go-Gn	20.1°	N	15°	20°	25°	0°
S-N^sna-snp	11.6°	N	7°	10°	13°	0°
S-N^PO	17.3°	x	11°	14°	17°	0.3°
S-N^Go-Gn	31.7°	N	27°	32°	37°	0°
SNBa	140.3°	xx	124°	129°	134°	6.3°
SND	80.9°	xx	74°	76°	78°	2.9°
IS^II	135.3°	x	125°	130°	135°	0.3°
IS:N-A	2.7	−x	3	4	5	0.3
II:N-B	3.5	N	3	4	5	0
II:A-Pog	3.2	x	−1	1	3	0.2
Ls:Line S	−3.9	−xxx	−1	0	1	2.9
Li: Line S	−4.4	−xxxx	−1	0	1	3.4
Cvm:S-Gn	−3.6	−xxx	−1	0	1	2.6
Mol Sup^P. Occl	108°	xxx	88°	90°	92°	16°
N-S-Cop	137°	xxx	117°	122°	127°	10°
S-Cop-Go	128.7°	−xx	137°	143°	149°	8.3°
Cop-Go-Gn	126.1°	x	115°	120°	125°	1.1°
Cop-Go-N	54.4°	xx	48°	50°	52°	2.4°
N-Go-Gn	71.6°	N	68°	70°	72°	0°
II^Go-Gn	79°	−xxx	92°	93°	94°	13°
SOr:sna	63.3		0	0	0	63.3
sna:Me	57.5		0	0	0	57.5
S:N	59.4	−xxxx	68.7	71.7	74.7	9.3
snp:A	49.7		0	0	0	49.7
Go:Me	74.6	N	66.7	71.7	76.7	0
Wits	−7.8	−xxx	−2	0	2	5.8
IS^N-S	113.9°	xxxxx	101°	103°	105°	8.9°
Pog:N-B	0.3		0	0	0	0.3
Pog:N-B—II:N-B	−3.2	-	0	0	0	3.2

**Table 5:** Cephalometric analysis before treatment.

Cephalometric Analysis before Treatment	Val	Dev	Min	Med	Max	Diff
SNA	82.4°	N	80°	82°	84°	0°
SNB	74.7°	−xx	78°	80°	82°	3.3°
ANB	7.7°	xx	0°	2°	4°	3.7°
sna-snp^Go-Gn	23.2°	N	15°	20°	25°	0°
S-N^sna-snp	10.6°	N	7°	10°	13°	0°
S-N^PO	20.6°	xx	11°	14°	17°	3.6°
S-N^Go-Gn	33.7°	N	27°	32°	37°	0°
SNBa	127.1°	N	124°	129°	134°	0°
SND	71.1°	−xx	74°	76°	78°	2.9°
IS^II	103°	−xxxxx	125°	130°	135°	22°
IS:N-A	2.9	−x	3	4	5	0.1
II:N-B	4.8	N	3	4	5	0
II:A-Pog	0.8	N	−1	1	3	0
Ls:Line S	2.6	xx	−1	0	1	1.6
Li:Line S	0.6	N	−1	0	1	0
Cvm:S-Gn	−3.3	−xxx	−1	0	1	2.3
Mol Sup^P. Occl	109°	xxx	88°	90°	92°	17°
N-S-Cop	118.3°	N	117°	122°	127°	0°
S-Cop-Go	156.4°	xx	137°	143°	149°	7.4°
Cop-Go-Gn	119°	N	115°	120°	125°	0°
Cop-Go-N	48.5°	N	48°	50°	52°	0°
N-Go-Gn	70.5°	N	68°	70°	72°	0°
II^Go-Gn	104.1°	xxx	92°	93°	94°	10.1°
SOr:sna	57.5		0	0	0	57.5
sna:Me	54.1		0	0	0	54.1
S:N	63.2	−xxx	71	74	77	7.8
snp:A	44.4		0	0	0	44.4
Go:Me	59	−xxx	69.3	74.3	79.3	10.3
Wits	4.2	xx	−2	0	2	2.2
IS^N-S	119.1°	xxx	101°	103°	105°	14.1°
Pog:N-B	1.6		0	0	0	1.6
Pog:N-B—II:N-B	−3.2	-	0	0	0	3.2

**Table 6:** Cephalometric analysis after treatment.

Cephalometric Analysis after Treatment	Val	Dev	Min	Med	Max	Diff
SNA	79.5°	−x	80°	82°	84°	0.5°
SNB	76.4°	−x	78°	80°	82°	1.6°
ANB	3.2°	N	0°	2°	4°	0°
sna-snp^Go-Gn	23.3°	N	15°	20°	25°	0°
S-N^sna-snp	11.4°	N	7°	10°	13°	0°
S-N^PO	20.1°	xx	11°	14°	17°	3.1°
S-N^Go-Gn	34.7°	N	27°	32°	37°	0°
SNBa	136.4°	x	124°	129°	134°	2.4°
SND	72.1°	−x	74°	76°	78°	1.9°
IS^II	122°	−x	125°	130°	135°	3°
IS:N-A	4	N	3	4	5	0
II:N-B	3.5	N	3	4	5	0
II:A-Pog	2.1	N	−1	1	3	0
Ls:Line S	−2	−xx	−1	0	1	1
Li:Line S	0	N	−1	0	1	0
Cvm:S-Gn	−0.1	N	−1	0	1	0
Mol Sup^P. Occl	95.8°	xx	88°	90°	92°	3.8°
N-S-Cop	129.4°	x	117°	122°	127°	2.4°
S-Cop-Go	141.5°	N	137°	143°	149°	0°
Cop-Go-Gn	123.8°	N	115°	120°	125°	0°
Cop-Go-N	52.5°	x	48°	50°	52°	0.5°
N-Go-Gn	71.3°	N	68°	70°	72°	0°
II^Go-Gn	99.9°	xxxxxx	92°	93°	94°	5.9°
SOr:sna	57.1		0	0	0	57.1
sna:Me	56.3		0	0	0	56.3
S:N	62.9	−xxx	71	74	77	8.1
snp:A	46.7		0	0	0	46.7
Go:Me	61	−xx	69.3	74.3	79.3	8.3
Wits	−0.8	N	−2	0	2	0
IS^N-S	103.4°	N	101°	103°	105°	0°
Pog:N-B	0.4		0	0	0	0.4
Pog:N-B—II:N-B	−3.1	-	0	0	0	3.1

**Table 7:** Cephalometric analysis before treatment.

Cephalometric Analysis before Treatment	Val	Dev	Min	Med	Max	Diff
SNA	74.5°	−xxx	80°	82°	84°	5.5°
SNB	70.3°	−xxxx	78°	80°	82°	7.7°
ANB	4.2°	x	0°	2°	4°	0.2°
sna-snp^Go-Gn	30.7°	xx	15°	20°	25°	5.7°
S-N^sna-snp	11.4°	N	7°	10°	13°	0°
S-N^PO	25.2°	xxx	11°	14°	17°	8.2°
S-N^Go-Gn	42.1°	xx	27°	32°	37°	5.1°
SNBa	140.5°	xx	124°	129°	134°	6.5°
SND	66.4°	−xxxx	74°	76°	78°	7.6°
IS^II	120.3°	−x	125°	130°	135°	4.7°
IS:N-A	1.9	−xx	3	4	5	1.1
II:N-B	2.2	−x	3	4	5	0.8
II:A-Pog	0.8	N	−1	1	3	0
Ls:Line S	0.9	N	−1	0	1	0
Li:Line S	−0.8	N	−1	0	1	0
Cvm:S-Gn	−3.3	−xxx	−1	0	1	2.3
Mol Sup^P. Occl	89.4°	N	88°	90°	92°	0°
N-S-Cop	135.2°	xx	117°	122°	127°	8.2°
S-Cop-Go	135°	−x	137°	143°	149°	2°
Cop-Go-Gn	131.9°	xx	115°	120°	125°	6.9°
Cop-Go-N	57.7°	xxx	48°	50°	52°	5.7°
N-Go-Gn	74.2°	xx	68°	70°	72°	2.2°
II^Go-Gn	94.3°	x	92°	93°	94°	0.3°
SOr:sna	52.9		0	0	0	52.9
sna:Me	57.3		0	0	0	57.3
S:N	63.7	−x	66.6	69.6	72.6	2.9
snp:A	41.6		0	0	0	41.6
Go:Me	57.7	−xx	66	71	76	8.3
Wits	0.1	N	−2	0	2	0
IS^N-S	103.4°	N	101°	103°	105°	0°
Pog:N-B	0.2		0	0	0	0.2
Pog:N-B—II:N-B	−2	-	0	0	0	2

**Table 8:** Cephalometric analysis after treatment and a 3-year follow-up period.

Cephalometric Analysis after Treatment	Val	Dev	Min	Med	Max	Diff
SNA	76.6°	−xx	80°	82°	84°	3.4°
SNB	74.1°	−xx	78°	80°	82°	3.9°
ANB	2.5°	N	0°	2°	4°	0°
sna-snp^Go-Gn	23.3°	N	15°	20°	25°	0°
S-N^sna-snp	12.7°	N	7°	10°	13°	0°
S-N^PO	25.7°	xxx	11°	14°	17°	8.7°
S-N^Go-Gn	36.1°	N	27°	32°	37°	0°
SNBa	138.2°	x	124°	129°	134°	4.2°
SND	71.2°	−xx	74°	76°	78°	2.8°
IS^II	130.3°	N	125°	130°	135°	0°
IS:N-A	1.6	−xx	3	4	5	1.4
II:N-B	2.3	−x	3	4	5	0.7
II:A-Pog	0.4	N	−1	1	3	0
Ls:Line S	−2	−xx	−1	0	1	1
Li:Line S	−1.8	−x	−1	0	1	0.8
Cvm:S-Gn	4.4	xxxx	−1	0	1	3.4
Mol Sup^P. Occl	92.1°	x	88°	90°	92°	0.1°
N-S-Cop	133.1°	xx	117°	122°	127°	6.1°
S-Cop-Go	133.6°	−x	137°	143°	149°	3.4°
Cop-Go-Gn	129.4°	x	115°	120°	125°	4.4°
Cop-Go-N	57.4°	xxx	48°	50°	52°	5.4°
N-Go-Gn	71.9°	N	68°	70°	72°	0°
II^Go-Gn	92.1°	N	92°	93°	94°	0°
SOr:sna	64.3		0	0	0	64.3
sna:Me	56.5		0	0	0	56.5
S:N	65.6	−x	67.3	70.3	73.3	1.7
snp:A	49.3		0	0	0	49.3
Go:Me	63.6	−x	66	71	76	2.4
Wits	−2.7	−x	−2	0	2	0.7
IS^N-S	101.6°	N	101°	103°	105°	0°
Pog:N-B	1.5		0	0	0	1.5
Pog:N-B—II:N-B	−0.8	-	0	0	0	0.8

**Table 9:** A schematic summary of traditional functional devices.

Passive Activators Myofunctional Activators Biodynamic Activators		Active Activators Biodynamic Activators
Rigid:	Bimaxillary:	- Bass
- Bionator	- Bassani appliance	- Lehman
- Andresen-Haulp	- Ducovator	- Teuscher
Elastic:	Double plates:	
- Bimler	-Twin Block	
- Frankel	- Planas	
- Cervera	- Kinetor	
- Elastodontic appliances	- Sanders	

## Data Availability

All experimental data to support the findings of this study are available contacting the corresponding author upon request.

## Abbreviations

AMCOP	Cranium-Occluded-Postural Multifunctional Harmonizers
EGA	Eruption Guidance Appliance
EF	Éducation Fonctionnelle
T4K	Trainer For Kids
POT	Pre-Orthodontic Trainer
MRC	Myofunctional Research
MFS	Multi-Function System
TMJ	Temporo-Mandibular-Joint
OSAS	Obstruction Sleep Apnea Syndrome
CVM	Cervical Vertebral Maturation
CVMS	Cervical Vertebral Maturational Stages
EOT	Early Orthodontic Treatment
EA	Elastodontic Appliance
Co	Condylion
Gn	Gnathion
Me	Menton
